# Using aggregated data from Swedish national quality registries as tools to describe health conditions of older adults with complex needs

**DOI:** 10.1007/s40520-020-01629-6

**Published:** 2020-06-13

**Authors:** Linda Johansson, Deborah Finkel, Christina Lannering, Anna K. Dahl Aslan, Boel Andersson-Gäre, Jenny Hallgren, Ulrika Lindmark, Marie Ernsth Bravell

**Affiliations:** 1 Institute of Gerontology, Aging Research Network – Jönköping(ARN-J), School of Health and Welfare, Jönköping University, Jönköping, Sweden; 2 Department of Psychology, Indiana University Southeast, New Albany, IN USA; 3 Region Jönköping County, Futurum, Ryhov, Jönköping, Sweden; 4 Department of Medical Epidemiology and Biostatistics, Karolinska Institutet, Stockholm, Sweden; 5 Jönköping Academy for Improvement of Health and Welfare, School of Health and Welfare, Jönköping University, Jönköping, Sweden; 6 School of Health Sciences, University of Skövde, Skövde, Sweden; 7 Department of Natural Science and Biomedicine, School of Health and Welfare, Centre for Oral Health and Aging Research Network – Jönköping (ARN-J), Jönköping University, Jönköping, Sweden

**Keywords:** National quality register, Older adults, Health care, Social service

## Abstract

**Background:**

Combining National Quality Registries (NQRs) with existing National Health Registries (NHRs) might make it possible to get a wider picture of older adults health situation. The aim was to examine the feasibility of aggregating data across different NQRs and existing NHRs to explore the possibility to investigate trajectories and patterns of disease and care, specifically for the most ill older adults.

**Method:**

A Swedish twin population (*N* = 44,816) was linked to nine NQRs and four NHRs. A descriptive mixed-method study was performed. A manifest content analysis identified which health parameters were collected from each NQR. Factor analysis identified patterns in representation across NQRs. Two case studies illustrated individual trajectories of care by using NQRs and NHRs.

**Results:**

About 36% of the population was registered in one or more NQRs. NQRs included 1849 variables that were sorted into 13 categories with extensive overlap across the NQRs. Health and function variables were identified, but few social or cognitive variables. Even though most individuals demonstrated unique patterns of multi-morbidities, factor analysis identified three clusters of representation in the NQRs with sufficient sample sizes for future investigations. The two cases illustrated the possibility of following patterns of disease and trajectories of care.

**Conclusions:**

NQRs seem to be a significant source for collecting data about a population that may be underrepresented in most research on aging because of their age and poor health. However, NQRs are primarily disease related, and further development of the registries to maximize coverage and utility is needed.

**Electronic supplementary material:**

The online version of this article (10.1007/s40520-020-01629-6) contains supplementary material, which is available to authorized users.

## Background

Even if most older adults in Sweden are currently living independently with good health and function, a majority will at some point before death be in need of health and social care [1]. This later stage of life can be described by broad losses in biological, functional and social abilities [2] but the trajectories towards multiple illnesses and functional impairments show a wide variation. Heterogeneity in developmental trajectories may go unnoticed if researchers implicitly rely on methods that endorse the assumption of uniform changes with age. Moreover, evidence shows that several conditions among older adults are associated, for example, dental plaque bacteria cause systemic inflammation and cardiovascular disease [3], nutritional status is linked to falls [4], and so on. Thus, it is important that assessments and treatments are based on an interdisciplinary approach and not only on extrapolations of research results from patients with single diseases [5]. Similarly, prevention and treatment guidelines for single diseases may be inappropriate when making care decisions for the most ill older individuals [6], i.e. individuals with complex impairments, since guidelines generally do not take the whole situation, including biological, psychological and social factors, for the older adults, into account [7]. A holistic approach can help health care providers to focus on areas most significant for the older adult, avoiding unnecessary treatments and adverse drug events [8].

The challenge facing health care systems is both to identify individuals before complex impairments become predominant and also to meet and treat individuals with complex impairments (called “the most ill older adults” in Sweden). Evidence suggests that the coordination of various care services and interventions for these most ill older adults is currently unsatisfactory [9, 10]. As practice re-organizes to meet the challenge of the most ill older adults, research on multimorbidity and multi-impairment needs to be strengthened and implemented in practice [11].

To improve health and social care for the most ill older adults, the Swedish government, therefore, decided in 2010 to invest 4.3 billion SEK (approximately $400 million). The overall goal of the effort was to improve care, support and treatment for these individuals and also to improve collaboration between different care and service settings and between different disciplines and professions [12]. In line with this effort, the Swedish government together with the Swedish Association of Local Authorities and Regions (SALAR) made an agreement in 2012 to further development National Quality Registries (NQRs). Prior to this agreement, quality registries existed but lacked sufficient support and therefore were often used primarily on a local level and initiated by single health care professionals, usually physicians [13]. Today, there are about 100 NQRs, and each NQR has its own steering committee involving professionals with expert knowledge in the area (some also include patients and informal caregivers) that are responsible for the development and content of the registry [14]. Most NQRs collect data regarding single conditions, medical treatment and outcomes. The main purpose of the recorded data is to monitor and compare adherence to guidelines and equality of care of the one disease, but data should also be used for quality improvement and research according to the vision from the government and SALAR [15, 16]. Sweden is fairly unique in the extensive number of NQRs it maintains; however, comparable registers do exist internationally regarding, for instance, stroke care [17], and from a global perspective the interest in further development of quality registers to improve patient safety and quality of care is high [18]. Studying the development and use of NQRs in Sweden can help to identify benefits as well as shortcomings, to provide an example for other countries. Another advantage of using Swedish data is that unique personal identification numbers have been in use since 1947, which enables researchers to collate extensive information from different registries on an individual level. Sweden also monitors health and social in National Health Registries (NHRs) like the National Patient Registry, the Prescribed Drug Registry, the Social Care Registry and the Cause of Death Registry, where all are obliged for health care staff to register.

Mattson [19] claims that combining different data sources can be an effective way to measure and develop health care for older adults, and it has previously been suggested that data from different registries should be used together in research. Combining registries in research is quite common and can provide data to follow patients throughout their lives [20–22], for instance, there are government-administered health registries focusing on hospital-based inpatient and outpatient care, prescribed medication, and cause of death. Emilsson and colleagues [15] indicated that several NQRs collect similar or identical data such as BMI, blood pressure, smoking and quality of life. However, little is known about experiences and effects of combining NQRs in research (or in practice) and whether it provides a wider picture of older adults’ health situation. Our aim is to examine the feasibility of aggregating data across different NQRs and existing NHRs to explore the possibility of understanding trajectories and patterns of disease and care, specifically for the most ill older adults. To our knowledge, this study represents the first attempt to leverage NQRs in this way.

Feasibility was examined by addressing these questions:

To what extent do older adults appear in the NQRs?

What function and health parameters are available in the NQRs and how much overlap exists in these variables across the NQRs?

Description of health conditions was explored by addressing these questions:

Are there any patterns of individuals being recorded across the NQRs?

Is it possible to trace individual trajectories of care across the NQRs and NHRs?

## Method

### Design

A descriptive mixed-method study including both qualitative and quantitative analyses was performed.

### Participants and data collection

All individuals in the Swedish Screening Across Lifespan Twin Study (SALT) were used as the base population for the current study. SALT was a telephone survey that started in 1998 and ended in 2002. All twins born before 1958 were invited to participate and 44,816 accepted. The SALT population provided a means for selecting a large representative sample from the NQRs. SALT is also linked with some NHRs: The Cause of Death Registry, the National Patient Registry, the Prescribed Drug Registry and the Social Care Registry. The mean age for participating in SALT was 59.4 ± 11.1 (range 41–103), and 53.5% percent were women. Previous studies have demonstrated that twins in the Swedish Twin Registry are representative of the Swedish population in general [23, 24].

Receiving and linking NQR data to SALT started in 2014 and the process included several steps as described in Online Resource 1. The criteria for selecting which NQRs to include in the current study were that they should comprise mainly older adults and/or diseases common in late life, large populations, describe life style and health parameters, and have a reasonable national coverage level. The selected nine NQRs are: the Swedish Heart Failure Registry (RiksSvikt), Swedish Web-system for Enhancement and Development of Evidence-based care in Heart disease (Swedeheart), Swedish Stroke registry (Riks-Stroke), Swedish diabetes registry (NDR), The Swedish Rheumatology Quality Registry (SRQ), Swedish National Hip Fracture Registry (Rikshöft), Better Management of Patients with OsteoArthritis (BOA-registret), Swedish Dementia Registry (SveDem) and Senior Alert, a preventive quality register for older adults. Information about the NQRs is presented in Online Resource 2.

### Analyses

#### Content analysis

To identify which health parameters were collected from each NQR, a manifest inductive content analysis inspired by Berg and Lune [25] was performed. All variables were entered in a spreadsheet; similar variables were grouped into subcategories, which were then clustered into higher-order categories. The variables in each subcategory as well as category were examined to demonstrate the extent of measure overlap across NQRs.

#### Factor analyses

Membership in each NQR was indicated for each individual. Oblique factor analysis was applied to this data set to investigate whether individuals tended to cluster in the same set of NQRs. The resulting factor structure was then used to create groups of individuals and analysis of variance was used to compare demographic variables across these groups.

#### Case analysis

To illustrate individual trajectories of care across the NQRs two cases were used. Cases can give increased insight into the care of a typical patient as well as focusing on the care system, e.g., progression of care through various points of contact with the healthcare system [26]. The cases are described using data from SALT as well as the NQRs and the NHRs. From the NQRs and NHRs, all available data were used for the included cases between 2010 and 2014; however, some variables had missing data and there were no available data for 2013 in the Social Care Registry. A flow chart was used to track the care process through different registries. Two individuals were selected based on the criteria of being “most ill” according to the definition by the Swedish government: 65 years or older, having an extensive need of medical care (either 3 diagnosis or more, at least 19 hospital days or, 3 hospitals stays) within the last 12 months as well as extensive care needs (receiving more than 25-h home-help per month, short-term or nursing home residents or receiving care in accordance with the Swedish act concerning support and service for individuals with certain functional impairments). In total, 161 older adults fit the inclusion criteria, and two cases were randomly chosen to illustrate individual trajectory.

## Results

### To what extent do older adults appear in the NQRs?

Among the participants in SALT (*N* = 44,816), 36.2% were registered in one or more NQRs, meaning that a majority (63.8%) was not registered in the selected NQRs. The distribution is further described in Table 1. The median number of NQRs, for those included in any NQR, was 1.0 (q1:1.0; q3:2.0) and the range was 1.0–6.0. Age and number of NQRs correlated at 0.16 (*p* < 0.001), indicating that older adults tended to appear in more NQRs.

**Table 1:** Distribution of the SALT population in the selected NQRs

Number of NQRs	*N* (%)
0	28,609 (63.8)
1	10,870 (24.3)
2	3955 (8.8)
3	1117 (2.5)
4	234 (0.5)
5	29 (0.1)
6	2 (0.0)

All the studied NQRs had someone registered from the SALT population. The most common NQR was Swedeheart (Swedish Web-system for Enhancement and Development of Evidence-based care in Heart disease) followed by Senior Alert (preventive quality register for older adults) and NDR (Swedish diabetes registry); over 5,000 individuals were registered in each. When combining across two NQRs the number of individuals decreases (see Table 2), and the highest number of individuals was found when combining across Swedeheart and Senior Alert (*N* = 1239), followed by Swedeheart and NDR (*N* = 1220).

**Table 2:** Representation of the SALT population (*n* = 44,816) across the NQRs

	Senior Alert	NDR	SRQ	SveDem	Swedeheart	BOA	Rikshöft	Riks-stroke	RiksSvikt
Senior Alert	5179								
NDR	1022	5109							
SRQ	71	68	539						
SveDem	330	120	0	645					
Swedeheart	1239	1220	71	109	5944				
BOA-registret	42	48	6	1	48	404			
Rikshöft	630	239	19	73	289	9	1803		
Riks-Stroke	810	533	26	92	720	10	269	2967	
RiksSvikt	204	181	4	20	356	4	54	93	634

*Senior Alert* preventive quality register for older adults, *NDR* Swedish diabetes registry, *SRQ* Swedish Rheumatology Quality Registry, *SveDem* Swedish Dementia Registry, *Swedeheart* Swedish Web-system for Enhancement and Development of Evidence-based care in Heart disease, *BOA-registret* Better Management of Patients with OsteoArthritis, *Rikshöft* Swedish National Hip Fracture Registry, *Riks-Stroke* Swedish Stroke registry, *RiksSvikt* Swedish Heart Failure Registry

### What function and health parameters are available in the NQRs and how much overlap in these variables exists across the NQRs?

In total, 1849 variables were included in the content analysis, sorted into 13 categories listed in the first column of Table 3 and 76 subcategories. Examples of the subcategories are provided in column 4 and column 5 indicates the number of NQRs that included each subcategory. The subcategories appear in an average of 4.73 NQRs (sd = 1.98), ranging from 2 NQRs for Home-help/Day care to 8 or 9 registries for basic information such as Background and Care Chain. For example, the Function/ADL subcategory represents 33 variables and is assessed via various methods in 5 NQRs: walking difficulty and use of assistant devices (BOA-registret [Better Management of Patients with OsteoArthritis] and Rikshöft [Swedish National Hip Fracture Registry]), require assistance with hygiene or dressing (Riks-Stroke [Swedish Stroke registry] and SveDem [Swedish Dementia Registry]), and staff assessment of mobility (SveDem, SRQ [The Swedish Rheumatology Quality Registry], and Riks-Stroke). As would be expected from quality registries that focus on health care, information about treatments is quite extensive (251 variables); whereas, information about cognitive function is typically limited to assessment of overall cognitive status or confusion (12 variables). The NQRs include an average of 11.56 (sd = 3.84) of the subcategories listed in column 2: RiksSvikt [the Swedish Heart Failure Registry] has the most subcategories at 16. Although Senior Alert assesses only 4 of the subcategories listed, the focus on care issues for older adults means that it is one of only two NQRs that contains extensive information about nursing care, including falls, nutrition, pressure ulcers, and oral health. Overall, there is extensive overlap among the NQRs at the level of categories and subcategories, allowing for careful combination of data across NQRs to examine, for example, body mass index (BMI) and quality of life, as well as the trajectory of care across NQRs.

**Table 3:** Results of content analysis of variables included in each NQR

Category	# of sub-categories	# of variables	Example subcategories	Number of NQRs	Riks Svikt	Swede heart	Riks-Stroke	NDR	SRQ	Rikshöft	BOA-registret	Svedem	Senior Alert
Background	2	19	Age and sex	8	X	X	X		X	X	X	X	X
Care chain	1	108	Admit/discharge/follow-up	9	X	X	X	X	X	X	X	X	X
Death	1	10	Date/Time	5	X	X	X			X		X	
Diagnoses	8	226	Various diagnoses	7	X	X	X	X	X	X		X	
Health	8	304	Function/ADL	5			X		X	X	X	X	
			Physical activity	3		X		X			X		
			Quality of life	6	X	X			X	X	X	X	
			Alcohol/tobacco	6	X	X	X	X		X	X		
			General/fatigue/Sensory	3	X		X		X				
Exams	3	18	ECG/X-ray	3	X	X					X		
Lab tests	12	50	Lipids/glucose/other	4	X	X		X	X				
Measures	6	89	Height/weight	7	X	X		X		X	X	X	X
			BP	3	X	X		X					
Mental health	4	123	Coping/anxiety/depression	3	X		X				X		
			Cognition	3			X			X		X	
Nursing	7	152	Falls/nutrition/pressure ulcers/oral health	2			X						X
Social	8	264	Education/occupation	4		X			X		X	X	
			Family/housing/marital status	5	X		X			X	X	X	
			Home-help/day care	2			X					X	
Symptoms	3	235	Joints/cardiovascular/pain	5	X	X			X	X	X		
Treatment	13	251	Medications	7	X	X	X	X		X	X	X	
			Operations	4	X	X				X	X		
TOTAL	76	1849	Subcategories per NQR		16	15	13	8	9	13	14	12	4

*Senior Alert* preventive quality register for older adults, *NDR* Swedish diabetes registry, *SRQ* Swedish Rheumatology Quality Registry, *SveDem* Swedish Dementia Registry, *Swedeheart* Swedish Web-system for Enhancement and Development of Evidence-based care in Heart disease, *BOA-registret* Better Management of Patients with OsteoArthritis, *Rikshöft* Swedish National Hip Fracture Registry, *Riks-Stroke* Swedish Stroke registry, *RiksSvikt* Swedish Heart Failure Registry

### Are there any patterns of individuals being recorded across the NQRs?

Factor analysis resulted in a three-factor solution that accounted for a total of 41% of the variance. Factor loadings and factor inter-correlations are presented in Table 4. Descriptive statistics for the groups are presented in Online Resource 3. The factor analysis demonstrated that there was little overlap in the factor loadings defining the factors: factor 1 included NQRs that track stroke, dementia, hip fractures, and aging issues (Senior Alert); factor 2 included NQRs that tap heart-related health issues; and factor 3 tapped forms of arthritis. Groups were created based on the results of the factor analysis. One-way ANOVA indicated significant differences in birth years among the groups (*F*(4, 18,278) = 272.42, *p* < 0.01). Group 1 had the lowest mean birth year (1928) and Group 3 had the highest mean birth year (1944).

**Table 4:** Factor analysis of NQRs representation

NQR	Factor 1	Factor 2	Factor 3
The social care registry	**0.78**	0.09	0.00
Senior alert	**0.69**	0.12	0.05
Riks-Stroke	**0.32**	0.19	− 0.09
Rikshöft	**0.54**	− 0.17	0.02
SveDem	**0.49**	− 0.16	− 0.07
Swedeheart	− 0.03	**0.71**	0.00
NDR	0.02	**0.51**	0.10
RiksSvikt	− 0.06	**0.63**	− 0.06
SRQ	0.01	− 0.01	**0.79**
BOA-registret	− 0.05	0.02	**0.59**
% variance explained	17.67%	13.36%	10.05%
Intercorrelations			
Factor 2	− 0.19		
Factor 3	− 0.01	0.01	

Factor loadings greater than 0.30 are in bold face

*Senior Alert* preventive quality register for older adults, *NDR* Swedish diabetes registry, *SRQ* Swedish Rheumatology Quality Registry, *SveDem* Swedish Dementia Registry, *Swedeheart* Swedish Web-system for Enhancement and Development of Evidence-based care in Heart disease, *BOA-registret* Better Management of Patients with OsteoArthritis, *Rikshöft* Swedish National Hip Fracture Registry, *Riks-Stroke* Swedish Stroke registry, *RiksSvikt* Swedish Heart Failure Registry

### Is it possible to trace individual trajectories of care across the NQRs?

**Fig. 1:**
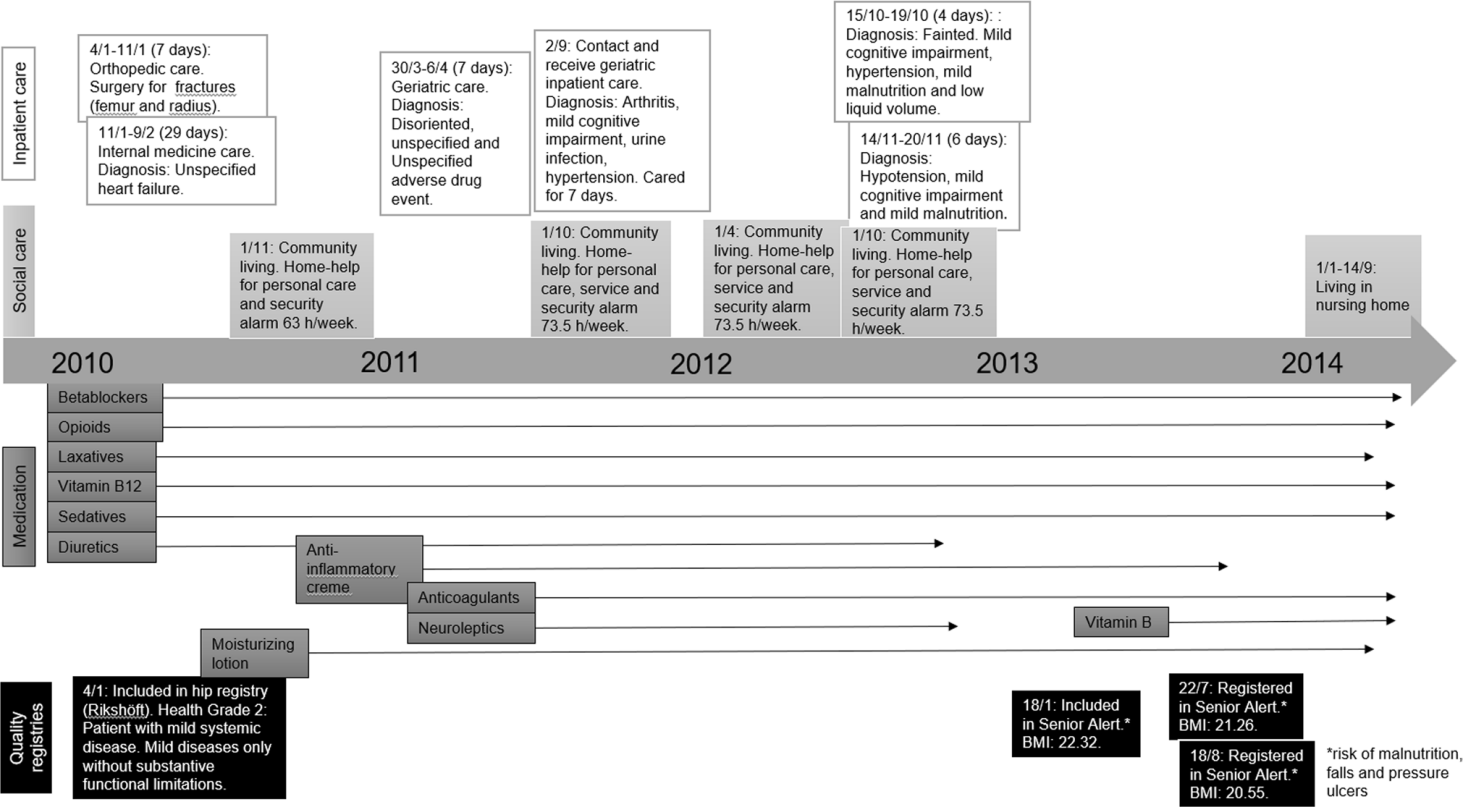
Flow chart of registration for case A

Case A is a woman born in 1915 (Fig. 1). According to the National Patient Registry, she fell in 2010 and suffered from fractures. She was then registered in the hip fracture registry (Rikshöft) and assessed as having other mild illnesses with mild substantive functional limits. Already in 2010, the woman received more than five different medications including opioids, sedatives and beta blockers (the Prescribed Drug Registry). In 2011, she was diagnosed with mild cognitive impairment according to the National Patient Registry. During the same inpatient care period, she was also diagnosed with unspecified adverse effects of drugs and medicines. Later the same year, she started to take neuroleptics and occasionally received additional pain medications (the Prescribed Drug Registry). Data from the Social Care Registry revealed that she was living in her home and received home-help service between 2010 and 2012. Initially, she had 63 h/week of home-help service and over time, it increased slightly to over 70 h/week including personal care, service, and a security alarm. It was not until 2013 she was registered in Senior Alert and assessments indicated that she was at risk for malnutrition, pressure ulcers, and falls. Also, her BMI successively decreased from 22.32 in January 2013 to 20.55 in August of the same year. She moved to a nursing home at the end of her life (the Social Care Registry) and passed away in September 2014 at the age of 99 (the Cause of Death Registry).

**Fig. 2:**
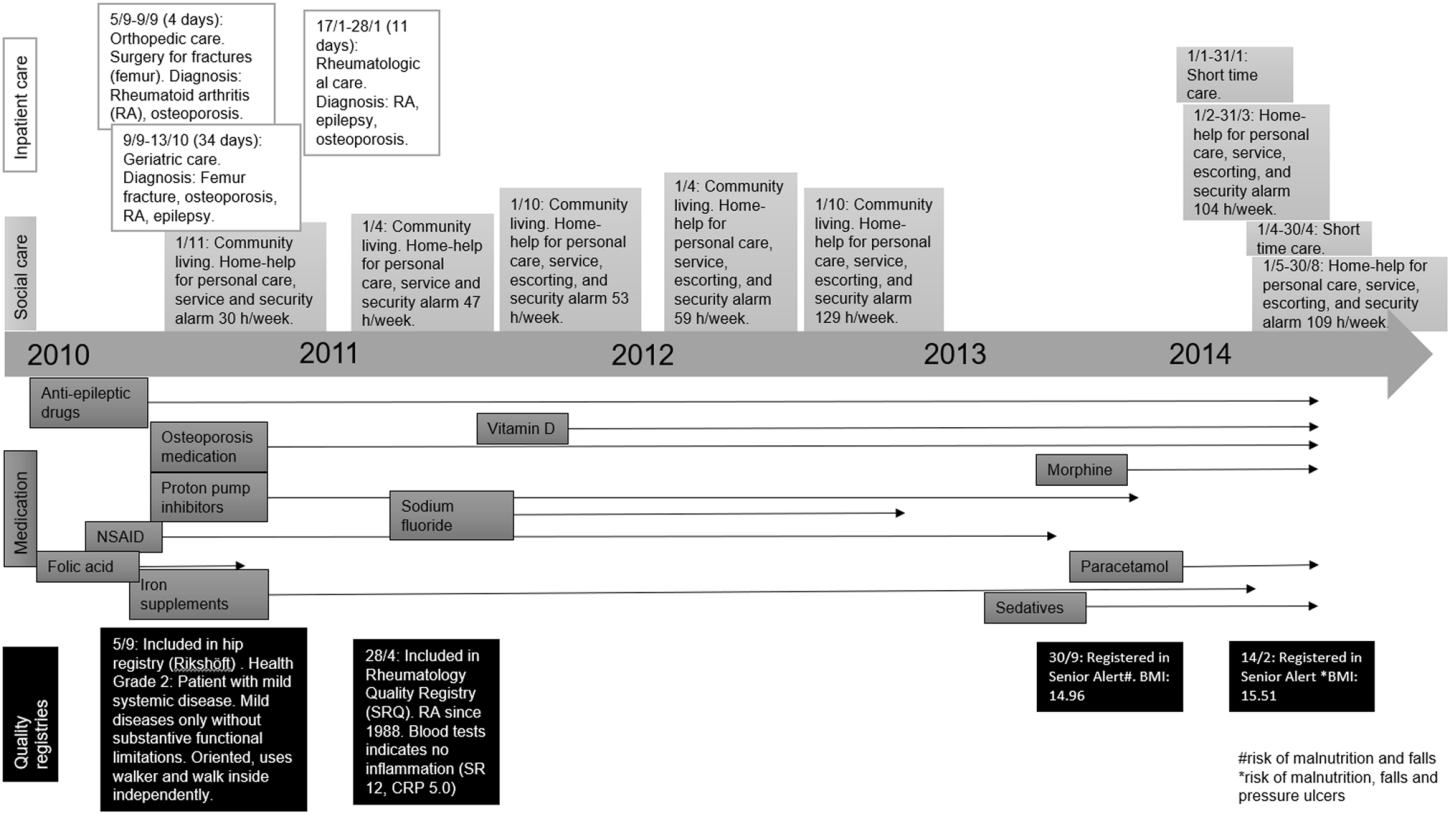
Flow chart of registration for case B

Case B is a man born in 1939 (Fig. 2). In September 2010, he was cared for in the hospital because of fractures and at the same time diagnosed with rheumatoid arthritis, epilepsy, and osteoporosis (the National Patient Registry). During this inpatient care, he also started medication for osteoporosis (the Prescribed Drug Registry) and was registered in Rikshöft (Hip Fracture Registry). According to the health assessment performed in Rikshöft, he was considered to have mild illnesses with mild substantive functional limits. In 2011, he was registered in the rheumatology registry, SRQ. During 2011 and 2012 he received inpatient care only once, in January 2011. However, he had contact with several specialists because of his medical conditions, including specialists in rheumatology and internal medicine (the National Patient Registry). In 2013, he stopped taking the NSAID and later that year, he first started taking sedatives and then also opioids (the Prescribed Drug Registry). The Social Care Registry indicates that he was living in his home with support from home-help care. In 2010, he received 30 h/week of home-help care; while in 2014, he received over 100 h/week and, in turn, he also received short-term residential care, for example for rehabilitation. According to the Senior Alert registrations in 2013 and 2014, he was considered at risk of malnutrition and was underweight (BMI 15 kg/m^2^) but did not receive any meal support at any time according to the data from the Social Care Registry. He passed away in September 2014 at the age of 75 (the Cause of Death Registry).

## Discussion

An important strength of the NQRs is the ability to collect data about a population that may be underrepresented in most research on aging because of their age and poor health. We note that increasing age was associated with registration in an increasing number of NQRs. To the extent that individuals have multimorbidities, they should be included in multiple NQRs. Factor analysis indicated that even though many individuals experienced unique patterns of multimorbidity, there were, indeed, patterns of representation in the NQRs for about 25% of the older adults. It is also possible that variations in recording levels for the NQRs hindered our ability to detect larger patterns of membership across NQRs. Even so, there are sufficient numbers of older adults represented in clusters of NQRs that focus on conditions of the older population (group 1), heart conditions (group 2), or arthritis (group 3) to allow for future analyses of the patterns of care of individuals in these groups. The two case studies could be extended to the larger sample to examine the effectiveness of care for particular conditions.

Some of the NQRs included many health and function variables and there is considerable overlap among the NQRs at the level of higher-order variable categories and subcategories allowing for combination of data across NQRs to investigate some variables, for example, BMI or quality of life or trajectories of care. However, there are limited measures of social or cognitive factors which again may be related to the fact that NQRs are mainly disease related [15]. In contrast, Senior Alert (the preventive quality register for older adults) includes information about care and is a unique source of information as it focuses on areas of importance for gerontological nursing care. The areas include malnutrition, falls, pressure ulcers, and oral health, all of which are highly related to frailty [27, 28]. These types of assessments are of paramount importance in efforts to improve care as well as health and well-being in older adults with complex needs. From the Senior Alert alone, however, it is not possible to extract any other information about the individuals, such as diseases or quality of life, highlighting the need to combine information across NQRs.

In total, about one-third of the participants in SALT were registered in an NQR. Some NQRs may describe important health aspects among older adults, but others have too few registered individuals for the purpose, i.e., the completeness and coverage rate are low. For most NQRs, the completeness rate can be demonstrated by analyzing the number of individuals with certain diagnoses in the National Patient registry (an obligatory registry), and matching it with the completeness of the NQR [29]. The completeness and registration can be influenced by several things and depends, for instance, on how long an NQR has been used [30]. Completeness and coverage are also related to where care is given. According to National Board of Health and Welfare the number of older adults receiving home-care nursing is increasing but few NQRs focus on describing the care given in that context, instead the focus is on health care in hospitals or primary care [31]. Also, as the mean age for being included was rather high, it is likely that some individuals in SALT passed away before an NQR was developed. All together, this affects interpretation of the results, since it is unclear how representative an NQR is of all possible cases.

Consequently, it can be difficult to describe health and individual trajectories from a broader perspective and with a focus on older adults and diseases common in later life by using information from the currently existing NQRs. However, in recent years, patient-reported outcomes measures (PROM), such as self-rated health and quality of life, have increasingly been included in NQRs [5, 32]. Such data were not available for the present study, but in the future, they may help to get a broader, more comprehensive perspective of older adults with complex needs. Also, the two case studies indicated that by combining several health registries with NQRs on an individual basis it is possible, to follow an individual over time and describe care in the last years in life. The two cases revealed a complex situation where care was given both from the municipality and the county council, i.e., including several care providers. The cases highlight not only the necessity of an interdisciplinary approach to meet the needs of these individuals [5] but also that good collaboration between hospital and primary care and the county council and municipality is required to provide the best possible care.

Finally, the many different individuals entering the data in the NQRs make them difficult to validate. In other words, even if the variables exist in the NQRs to describe health, it is important to consider whether the available data are valid. At the same time, data from NQRs are supposed to be entered routinely by health care staff; therefore, NQRs could provide important information without causing any additional distresses or discomfort among the most ill older adults. Further, our results show that interesting data and trajectories can be retrieved across the NQRs combined with NHRs. The study, thus, encourages further development of the NQRs with regard to completeness, coverage and use. For example, their direct linkage to electronic medical records is one factor that could ease the burden of entering data and at the same time create more valid data. A similar conclusion was made by Alguren et al. [33], who suggested that data management personnel could manage the administrative task to enter data, so that health care professionals could spend more time analyzing data and working with quality improvements.

## Conclusion

The information available in NQRs provides a starting point for the possible use of registry data as a basis for research on interventions into the issues associated with aging and particularly research on care for older adults. When used appropriately, NQRs provide a population-based representative network that has immediate external validity and established follow-up procedures. There is a fair overlap of functional and health parameters, such as body mass index and quality of life, among the NQRs allowing for combining data across NQRs to examine. Thus, the current analyses hint at the promise of NQR-based research to support care planning in older adults, if the NQRs are used and maintained consistently and combined strategically. There are several limitations of combining data from NQRs and use for research purposes. Consequently, further development is needed, for example, to minimize double recording as well as maximize coverage and utility. A take home message for countries aiming to develop NQRs would be to think carefully about which variables to include and that perhaps an overarching system can be more fruitful when collecting information to monitor and evaluate care as well as in quality improvement work and research.

## Electronic supplementary material

Below is the link to the electronic supplementary material.

Supplementary material 1 (DOCX 31 kb)

## References

[1] Ernsth Bravell M, Malmberg B, Berg S (2010). End-of-life care in the oldest old. Palliat Support Care.

[2] Baltes PB, Smith J (2003). New frontiers in the future of aging: from successful aging of the young old to the dilemmas of the fourth age. Gerontology.

[3] Carrizales-Sepúlveda EF, Ordaz-Farías A, Vera-Pineda R, Flores-Ramírez R (2018). Periodontal disease, systemic inflammation and the risk of cardiovascular disease. Heart Lung Circ.

[4] Westergren A, Hagell P, Sjödahl Hammarlund CJ (2014). Malnutrition and risk of falling among elderly without home-help service–a cross sectional study. Nutr Health Aging.

[5] Nilsson G (2012). Multisjuklighet är mer regel än undantag. Men forskningen har inte hängt med i dagens vårdverklighet [Multimorbidity is more of a rule than exception. But research has not kept up with today’s care reality]. Läkartidningen.

[6] Boyd CM, Darer J, Boult C, Fried LP, Boult L, Wu AW (2005). Clinical practice guidelines and quality of care for older patients with multiple comorbid diseases: implications for pay for performance. JAMA.

[7] Tkatch R, Musich S, MacLeod S, Alsgaard K, Hawkins K, Yeh CS (2016). Population health management for older adults: review of interventions for promoting successful aging across the health continuum. Gerontol Geriatr Med.

[8] WHO (2017). Integrated care for older people: guidelines on community-level interventions to manage declines in intrinsic capacity.

[9] Banerjee S (2015). Multimorbidity—older adults need health care that can count past one. The Lancet.

[10] WHO (2015). WHO global strategy on people-centred and integrated health services: interim report.

[11] Smith SM, Soubhi H, Fortin M (2012). Interventions for improving outcomes in patients with multimorbidity in primary care and community settings. Cochrane Database Syst Rev.

[12] Comprehensible health and care for the most ill elderly 2013. Ministry of Social Affairs [Government report # 2012-12-13 S 2012/8765/FST], Stockholm, Sweden (2012)

[13] Satsningen på Nationella Kvalitetsregister 2012–2016 [Investment in the National Quality Register 2012–2016]. (2017) [cited 2020 May 15]. http://kvalitetsregister.se/tjanster/omnationellakvalitetsregister/satsning20122016.2009.html

[14] Lapptäcke med otillräcklig täckning Slututvärdering av satsningen på nationella kvalitetsregister [Patchwork with insufficient coverage: Final evaluation of investment in national quality registers]. Myndigheten för vård- och omsorgsanalys [Authority for Health and Care Analysis], Stockholm, Sweden 2017

[15] Emilsson L, Lindahl B, Köster M, Lambe M, Ludvigsson JF (2015). Review of 103 Swedish healthcare quality registries. J Intern Med.

[16] Jacobsson Ekman G, Lindahl B, Nordin A (2015). Nationella kvalitetsregister i hälso- och sjukvården [National quality registers in health care].

[17] Cadilhac DA, Kim J, Lannin NA (2016). National stroke registries for monitoring and improving the quality of hospital care: a systematic review. Int J Stroke.

[18] McNeil JJ, Evans SM, Johnson NP, Cameron PA (2010). Clinical-quality registries: their role in quality improvement. Med J Aust.

[19] Mattsson T (2016). Quality registries in Sweden, healthcare improvements and elderly persons with cognitive impairments. Eur J Health Law.

[20] Asplund K, Glader EL, Norrving B, Eriksson M, Collaboration Riks-Stroke (2011). Effects of extending the time window of thrombolysis to 4.5 hours: observations in the Swedish stroke register (risk-stroke). Stroke.

[21] Religa D, Spångberg K, Wimo A, Edlund AK, Winblad B, Eriksdotter-Jönhagen M (2012). Dementia diagnosis differs in men and women and depends on age and dementia severity: data from SveDem, the Swedish Dementia Quality Registry. Dement Geriatr Cogn Disord.

[22] Chatzidionysiou K, Kristensen LE, Eriksson J, Askling J, van Vollenhoven R, ARTIS Group (2015). Effectiveness and survival-on-drug of certolizumab pegol in rheumatoid arthritis in clinical practice: results from the national Swedish register. Scand J Rheumatol.

[23] Lichtenstein P, Sullivan PF, Cnattingius S (2006). The Swedish Twin Registry in the third millenium: an update. Twin Res Hum Genet.

[24] Magnusson PK, Almqvist C, Rahman I (2013). The Swedish Twin Registry: establishment of a biobank and other recent developments. Twin Res Hum Genet.

[25] Berg BL, Lune H (2014). Qualitative research methods for the social sciences.

[26] Vu MH, Weinberg G (2018). Making the Case for Case Reports. Anesth Analg.

[27] Batchelor P (2015). The changing epidemiology of oral diseases in the elderly, their growing importance for care and how they can be managed. Age Ageing.

[28] Ernsth Bravell M, Westerlind B, Midlöv P (2011). How to assess frailty and the need for care? Report from the Study of Health and Drugs in the Elderly (SHADES) in community dwellings in Sweden. Arch Gerontol Geriatr.

[29] National Board of Health and Welfare Rapporteringen till nationella kvalitetsregister och hälsodataregistren Jämförelser av täckningsgrader 2014 [The reporting to national quality registers and health data registers. Comparisons of coverage rates 2014]. (2014) [cited 2020 May 15]. https://www.socialstyrelsen.se/globalassets/sharepoint-dokument/artikelkatalog/statistik/2014-12-7.pdf

[30] Lannering C (2018). Experiences and outcomes of systematic preventive work to reduce malnutrition, falls and pressure ulcers in nursing home residents, in School of Health and Welfare.

[31] National Board of Health and Welfare, 2017. Kvalitetsregister i kommunal hälso- och sjukvård En sammanställning baserad på samkörningar mellan kvalitetsregister och hälsodataregister [Healthcare Quality in public health A summary based on combined quality registers and health registers]. (2017). https://www.socialstyrelsen.se/globalassets/sharepoint-dokument/artikelkatalog/ovrigt/2017-1-25.pdf

[32] Registerdata för forskning [Registry data for research]. (2012) [cited 2020 May 15]. https://www.riksdagen.se/sv/dokument-lagar/dokument/statens-offentliga-utredningar/registerdata-for-forskning_H0B336

[33] Algurén B, Andersson-Gäre B, Thor J, Andersson AC (2018). Quality indicators and their regular use in clinical practice: results from a survey among users of two cardiovascular National Registries in Sweden. Int J Qual Health Care.

