# Supplementary material for: Using aggregated data from Swedish national quality registries as tools to describe health conditions of older adults with complex needs
Source: Aging Clin Exp Res. 2020 Jun 13;33(5):1297–306. doi: 10.1007/s40520-020-01629-6 (PMC8081709; doi:10.1007/s40520-020-01629-6)
Supplement: Supplementary file 1 — Supplementary material 1 (DOCX 31 kb) [file 40520_2020_1629_MOESM1_ESM.docx]

Online Supplement File for

Using aggregated data from Swedish national quality registries as tools to describe health conditions of older adults

Johansson, L., Finkel, D., Christina Lannering, Dahl Aslan, A., Andersson-Gäre, B., Hallgren, J., Lindmark, U., & Ernsth Bravell, M.

Resource 1: Description of method of retrieving data from National Quality Registries

Resource 2: Description of the NQRs used in the current analyses

Resource 3: Groups defined by the factor analysis of NQR representation.

Online Resource 1: Description of method of retrieving data from National Quality Registries

The process was not linear and required moving back and forth between the different steps: 1) Writing and receiving an ethical approval from the ethical research board in Linköping (dnr 2014/2635-271). 2) Establishing contact with staff at the National Board of Health and Welfare (NBHW) regarding how the data collection should be performed. 3) Searching for suitable National Quality Registries (NQRs) by examining variable lists and gathering information about the NQRs to evaluate whether they fit the inclusion criteria. Both the NQR’s own homepages and the NBHW homepage, as well as the review of NQR made by Emilsson and colleagues [1], were used for this step. Some NQRs fit most but not all inclusion criteria; in those cases the researchers discussed pros and cons of adding the registry until agreement was reached. 4) Contacting and writing applications to each NQR for data withdrawal including the NBHW which is responsible for the registry for the care and social services for the older adults and for individuals with impairments (the Social Care Registry). Data for all years a registry had been used until 2014 were collected. 5) An application was also sent to the twin registry for ID and data for the SALT-population. 6) After giving their approval the twin registry as well as the NQRs sent their data to the NBHW where all data were combined based on the person’s identification numbers. 7) Finally, the NBHW combined and anonymized all data and then sent it to us.

1. Emilsson, L., et al., *Review of 103 Swedish Healthcare Quality Registries.* J Intern Med, 2015. **277**: p. 94-136.

2. *National Board of Health and Welfare Rapporteringen till nationella kvalitetsregister och hälsodataregistren Jämförelser av täckningsgrader 2014 [The reporting to national quality registers and health data registers. Comparisons of coverage rates 2014]*. 2014 [cited 2020 May 15]; Available from: <https://www.socialstyrelsen.se/globalassets/sharepoint-dokument/artikelkatalog/statistik/2014-12-7.pdf>.

| **Online Resource 2. Description of the National Quality Registries (NQRs) used in the current analyses** | | | | | | |
| --- | --- | --- | --- | --- | --- | --- |
| **NQRs**  **(Swedish short name)** | **Purpose** | **Launched** | **Completeness (for data 2014)** | **Coverage (participating units/eligible units)** | **Volume per year** | **Users** |
| Swedish Heart Failure Registry  (RiksSvikt) | To improve treatment of patients with heart failure and, identify changes in the quality of care and content over time in county councils and health care units. | 2003 | 54,3%^1^.  Coverage based on main diagnosis of heart failure during inpatient care and a performed echocardiography, only centers with more than 10 registered patients are included. | 63/84^3^ | 9304^1^ persons | Registration during acute care, outpatient care/primary care and after 12 months.  Used by hospitals and outpatient care. |
| Swedish Web-system for Enhancement and Development of Evidence-based care in Heart disease  (Swedeheart) | To support the development of evidence-based therapy in acute and chronic coronary artery disease and in catheter-based or surgical valve intervention, and to detect changes in the quality and content of care over time in order to contribute to the advancement of risk prediction tools and decision support, as well as support continuous improvement efforts in all participating units and also to be used in research. | 2009. Four already established registries were merged together. Swedish Registry of Information and Knowledge about Swedish Heart Intensive Care Admissions (RIKS-HIA) was launched in 1995. Swedish Coronary Angiography and Angioplasty Registry (SCAAR) was launched in 1998. Heart surgery registry was launched in 1992. Secondary Prevention after Heart Intensive Care Admission (SEPHIA) is part of the RIKS-HIA. | Coverage for each of the four included registries:  RIKS-HIA^2^ 84% (lower among those <80 years old). Based on patient registry data  SCAAR^2^ 99%. Based on patient registry data  Heart surgery registry^2^ 96%. Based on patient registry data  SEPHIA^1^ 74% (measured 6-10 weeks and 12-14 months after registration in RIKS-HIA) | 71/71^3^ | 76000^3^ number of registrations, not unique persons. | Used by hospitals and outpatient care. |
| Swedish Stroke registry (Riks-Stroke) | A tool for continuous quality improvement of stroke care. The aim of the registry is to support high and consistent quality of care for stroke patients throughout Sweden, ultimately to ensure patient benefit in the form of the best possible care. | 1994 | 90% among first time stroke patients and 85% among persons diagnosed with a stroke^2.^ Based on patient registry data. | 72/72^3^ | 23562^1^ persons | All hospitals in Sweden admitting patients with acute stroke |
| Swedish diabetes registry (NDR) | To promote evidence-based development of diabetes care by offering up-to-date information about changes in the treatment of glycaemia and other risk factors, as well as diabetic complications. Another aim is to support improvement in the quality of care provided by participating units at hospitals and primary care clinics. The overall objective is to reduce morbidity and mortality, as well as to maximize the cost-effectiveness of diabetes care. | 1996 | 91% for those with drug treatment for the diabetes^2^. Based on drug treatment registry data. | 1268/1300^1^ | 368577^1^ persons | Hospitals and primary care |
| The Swedish Rheumatology Quality Registry (SRQ) | To continually improve the treatment and follow-up of patients with rheumatic diseases. | 1996 | Coverage 83%^2^. Based on patient registry data and drug treatment data. | 66/66^3^ | 49000^3^ persons | 56 different clinics for rheumatology all over the country enter health data in the registry. |
| Swedish National Hip Fracture Registry (Rikshöft) | To report performance measures to compare and create consistent and high-quality care in the country. A base for local improvements at different hospitals. | 1988 | Coverage 88%^2^. Based on patient registry data. | 53/54^1^ | 14000^3^ persons | Hospitals. |
| Better Management of Patients with osteoArthritis (BOA-registret) | To evaluate patient-reported outcomes following an intervention – the Supported Osteoarthritis Self-Management Program | 2010 | 77%^1^ of those in education program for osteoarthrosis. | 222/400^3^ | 13252^1^ | Units in all counties with education program for osteoarthrosis. |
| Swedish Dementia Registry (SveDem) | To improve quality of diagnostics, treatment and care of patients with dementia disorders. | 2007 | 34%^1^ counted relative to expected incidence. (approximately 50% are registered in specialist care and 50% in primary care settings) | 61/62^1^ specialist units  880/1180^1^ primary care units | 8148^1^ | Specialist care, primary care, and municipalities (nursing homes) |
| Senior Alert | To ensure a preventative approach for persons over 65 years of age with care needs within the areas of   - falls - ulcers/pressure sores - malnutrition - oral health | 2010 | No known coverage. | 13700/15000^1^ | 334377 number of registrations, not unique persons. | All professionals and organizations in the field of health and social care. |

^1^According data from the yearly reports at the registry’s homepage. Based on data from 2014.

^2^According to NHBW [2].

^3^According to Emilsson et al [1].

Online Resource 3. Groups defined by the factor analysis of NQR representation.

| Group | Definition | N | Mean Birth year (SD) |
| --- | --- | --- | --- |
| 1 | People in at least 3 of the 5 NQRs in factor 1 | 1332 | 1928 (7.80) |
| 2 | People in at least 2 of the 3 NQRs in factor 2 | 1387 | 1936 (9.97) |
| 3 | People in either NQR in factor 3 | 891 | 1944 (7.98) |
| 4 | People represented in more than one factor | 153 | 1931 (7.58) |
| 5 | People represented in none of the factors ^a^ | 14520 | 1934 (11.43) |

^a^ People represented in the 9 NQRs included in the factor analysis, but not in groups 1 through 4.
